# Distribution patterns and driving factors of fruit tree richness in North China

**DOI:** 10.3389/fpls.2026.1807195

**Published:** 2026-05-01

**Authors:** Zihan Wang, Yong Wang, Guoxiang Ding, Daoming Zhu, Xiaomeng Wang

**Affiliations:** 1 School of Ecology and Applied Meteorology, Nanjing University of Information Science and Technology, Nanjing, China; 2 Anhui Public Meteorological Service Center, Hefei, China; 3 State Key Lab of Severe Weather, Chinese Academy of Meteorological Sciences, Beijing, China; 4 Reading Academy, Nanjing University of Information Science and Technology, Nanjing, China

**Keywords:** fruit tree richness, Geodetector, MaxEnt, North China, structural equation modeling

## Abstract

Under the current backdrop of increasingly variable climatic conditions, the distribution patterns and driving factors of fruit tree richness in North China remain insufficiently understood, yet fruit tree diversity is critical for agricultural biodiversity and regional food security, and systematic studies integrating both natural and anthropogenic drivers are lacking in this region. In this study, we integrated distribution data of 14 major fruit tree species with climate, terrain, soil, and anthropogenic variables, covering North China (Beijing, Tianjin, Hebei, Shanxi, Shandong, and Henan provinces, approximately 1.18 million km²). Potential suitable habitats were simulated using the MaxEnt model to estimate fruit tree richness, and the Geodetector together with structural equation modeling (SEM) was applied to disentangle the multi-factor driving mechanisms. The results indicated that: (1) the simulated distributions of the 14 fruit tree species achieve high accuracy, with most species exhibiting AUC values above 0.8; (2) high fruit tree richness in the North China region is mainly concentrated in hilly and mountainous areas such as the eastern Taihang Mountains, the Shandong Hills, and Jiaodong Peninsula; (3) among individual environmental contributions, population density, annual mean temperature and nighttime light most frequently rank among the top three contributors across 14 species, with anthropogenic and topographic variables jointly governing fruit-tree habitat suitability in North China; (4) the interaction between climate and anthropogenic was strongest, with anthropogenic factors showing both direct negative associations with fruit tree richness and indirect pathways via climate and soil, whereas climate is positively associated with fruit tree richness. These findings demonstrate that anthropogenic factors, rather than purely natural conditions, dominate the spatial patterns of fruit tree richness in this intensively managed agricultural region. This study enhances understanding of the distribution of fruit tree richness in North China, clarifies the multi-factor mechanisms shaping its spatial patterns, and provides a useful reference for understanding how multiple environmental and anthropogenic factors are associated with fruit tree distribution, which may inform future studies and region-specific management strategies.

## 1 Introduction

Fruit tree richness, as an important component of agricultural biodiversity, is not only related to the stability and adaptability of the fruit tree industry, but also profoundly affects the sustainable development of regional agricultural systems (Altieri, 1999). Under the background of global climate change and agricultural intensification, the diversity of fruit tree species and varieties is facing increasingly severe challenges, and its spatial pattern and formation mechanism urgently require in-depth investigation. In agricultural landscapes, fruit tree distribution and richness are strongly shaped by human activities, which reflect market demand, management intensity, and economic inputs, and further regulate environmental conditions relevant to fruit tree growth (Cornille et al., 2015). Such anthropogenic influences interact with natural factors, including terrain, climate, and soil. Terrain conditions modify regional light, hydrothermal patterns, and water-holding capacity (Lin et al., 2013; Zhang et al., 2022); climatic factors regulate growth and distribution ranges through temperature and precipitation (Huang et al., 2024); while soil properties affect fruit tree distribution via nutrient availability and water regulation (John et al., 2007). However, there is currently a lack of systematic research on the dynamics of fruit tree richness and its driving factors, especially studies that comprehensively consider both natural environmental conditions and anthropogenic factors, which makes it difficult for us to accurately grasp the response of resources to environmental change, and also difficult to provide a basis for variety optimization and regional layout, thereby weakening the stability and sustainability of the industry. As an important fruit tree production area in China, the study of the richness pattern and causes in North China has practical significance for planting optimization and improvement of agricultural system resilience (Turdieva et al., 2024). Based on this, this paper will systematically analyze the spatial distribution and driving mechanisms of major fruit trees in North China, in order to provide a scientific basis for agricultural policy-making, germplasm resource conservation and utilization, and further enhance the adaptability of the global agricultural system to future challenges (Christianah and Folarin, 2024).

In the 19th century, natural scientists represented by Alexander von Humboldt conducted field surveys and manually recorded the distribution of species in different natural environments, analyzing the relationship between species’ suitable habitats and natural conditions. At the beginning of the 20th century, ecologist Joseph Grinnell first proposed the concept of ‘niche’ and emphasized the stable relationship between species and the environment. Subsequently, the “n-dimensional niche” theory proposed by G. Evelyn Hutchinson in 1957 marked the transition of species suitable habitat research from qualitative description to quantitative modeling. At the end of the 20th century, scientists began to use remote sensing technology and GIS technology to analyze NDVI data to study the relationship between vegetation growth and environmental conditions. A study predicting the global distribution of the invasive ant Solenopsis geminata under climate change using the MaxEnt model (Lee et al., 2021), the studies on the potential distribution range of Quercus suber in Italy (Vessella and Schirone, 2013) and Pinus sylvestris in Europe (Durrant et al., 2016). In recent years, the research objects of many studies have mainly focused on medicinal economic tree species (such as Lycium barbarum (Shen et al., 2023), Podocarpus falcatus (Tesfamariam et al., 2022) or oil-bearing economic tree species (such as oil palm (Elaeis guineensis) (Zon et al., 2025), olive (Kassout et al., 2022)), which are still studies on the suitable habitat distribution of single tree species. With the increasing demand for ecological protection and forestry planning, studies on multi-tree species richness have gradually emerged. Existing studies include distribution modeling of 17 Miombo tree species in sub-Saharan Africa (Jinga and Ashley, 2019), as well as prediction of the global richness distribution of the drought-tolerant genus Nitraria L. (Lu et al., 2025). Compared with single tree species distribution studies, these studies more comprehensively simulate natural ecosystems and provide more integrated strategies for ecological conservation and forestry planning. This paper focuses on the main fresh fruit and nut economic tree species in North China, covering 14 fruit trees of important economic value; through analyzing the suitable habitats and richness of these fruit trees under current environmental conditions and anthropogenic influences, it fills the gap in multi-tree species, especially fruit tree richness research in North China. In addition, compared with existing multi-species studies, this study integrates MaxEnt, Geodetector, and SEM within a unified framework to jointly analyze richness patterns, factor interactions, and driving pathways, while explicitly incorporating anthropogenic factors into the mechanism analysis; it will not only help to understand the ecological adaptability of multi-tree species in this region, but also provide scientific basis for the regional planning of fruit tree planting and adaptation strategies to climate change.

Species distribution models (SDMs) are effective tools for generating distribution patterns of species diversity under current climatic conditions. In the 1980s, the Commonwealth Scientific and Industrial Research Organisation (CSIRO) of Australia developed the Bioclim model (Booth et al., 2014). In the 1990s, Carpenter et al. developed the Domain model, and Stockwell and Peters developed the GARP model (Sánchez-Flores, 2007). The MaxEnt model, based on the maximum entropy principle proposed by E. T. Jaynes, was first proposed by Phillips et al. in 2004 (Phillips et al., 2004; Phillips et al., 2006). Initially used for single species modeling, it has been increasingly applied in multi-tree species studies since 2010. Compared with the GARP (Genetic Algorithm for Rule-Set Prediction) model (Haase et al., 2021), the MaxEnt model shows better performance in simulation accuracy, has greater advantages in handling small datasets, and can provide reliable estimates of suitable habitats under small sample conditions (Li et al., 2020, 2022), which is very suitable for the suitable habitat simulation of small-sample species such as Ficus carica and Prunus armeniaca in this paper. Compared with other models such as Domain (Domain Model) and Bioclim (Bioclimatic Envelope Model), the MaxEnt model not only produces more stable results, but also can output the contribution of each climatic factor (Tang et al., 2021; Xu et al., 2023), providing a clearer interpretation for model prediction results.

In summary, systematic studies on multi-fruit tree richness are still insufficient, with most existing studies focusing on the distribution simulation of single fruit tree species, while comprehensive studies on multi-tree species fruit tree richness in North China are still lacking. At the same time, the MaxEnt model has been widely applied due to its efficient and stable suitable habitat simulation ability under small sample conditions. This study focuses on 14 major fruit tree species in North China. Using species distribution data and driving variables, the MaxEnt model was employed to estimate suitable habitats and subsequently calculate fruit tree richness distribution. The Geodetector method was then applied to analyze how natural and anthropogenic factors interactively influence fruit tree richness. Furthermore, structural equation modeling (SEM) was utilized to uncover the driving pathways of climatic, terrain, soil, and anthropogenic factors. Collectively, these findings offer scientific evidence for optimizing fruit tree cultivation, thereby enhancing regional contributions to global food security through improved resource allocation and sustainable agricultural management strategies.

## 2 Materials and methods

### 2.1 Overview of the study area

**Figure 1:**
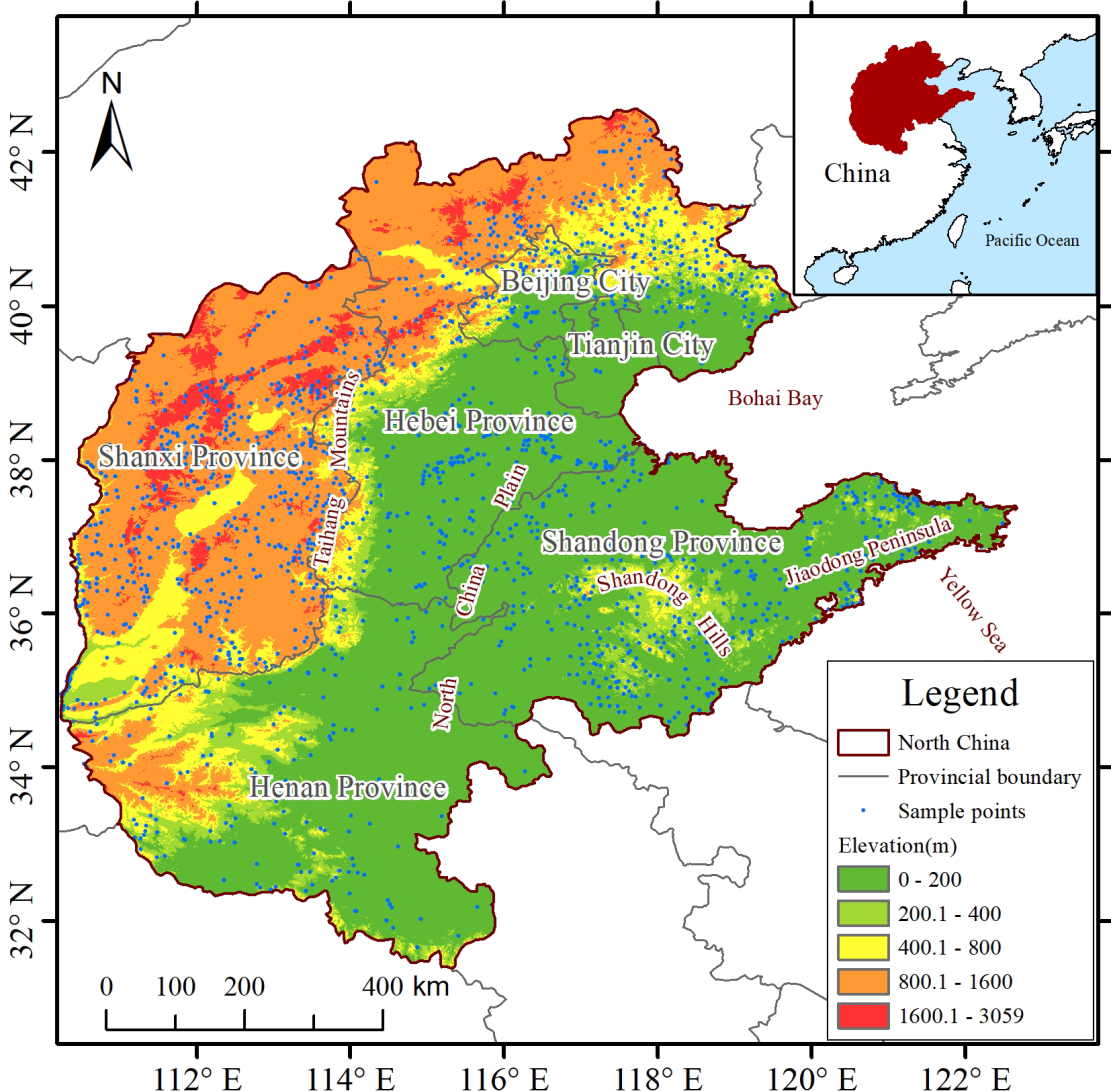
Overview map of the North China region.

North China is located between 110°15′–122°42′ E and 31°23′–42°40′ N, with a total area of about 1.1847 million km², including Beijing, Tianjin, Hebei Province, Shanxi Province, Shandong Province, and Henan Province (**Figure 1**). The region has complex and diverse terrain, gradually transitioning from the Inner Mongolia Plateau and the eastern edge of the Loess Plateau in the northwest to the low and flat North China Plain in the southeast, bordered by the Bohai Sea and the Yellow Sea to the east, and connected with the Qinling–Huaihe Line to the south, with the terrain sloping stepwise from northwest to southeast. This terrain gradient from plateau, hills, to plain not only shapes the spatial differences of environmental factors such as temperature, precipitation, and soil, but also directly affects the spatial distribution pattern of fruit tree richness (Badgley et al., 2017). The elevation of the North China Plain is mostly below 50 m, mainly formed by the alluvium of the Yellow River, Hai River, and Huai River channels, with sediment thickness reaching hundreds to thousands of meters, and it has extremely high farming potential. The region belongs to a warm temperate semi-humid continental monsoon climate with four distinct seasons: cold and dry winters, hot and rainy summers, mild springs and autumns with less precipitation, and frequent spring droughts. The annual precipitation ranges from 400–900 mm, showing a general trend of more in the south and less in the north, and concentrated in summer; the seasonal and regional imbalance of water resources significantly restricts agricultural production and imposes certain limitations on the distribution and diversity of fruit trees (Yang et al., 2021). North China is one of China’s oldest and most important agricultural belts, traditionally dominated by *Malus domestica*, *Pyrus pyrifolia*, *Prunus persica* and *Prunus armeniaca*. Under long-term land-use intensification that now includes high population density, dense road networks, bright nighttime-light indices and elevated GDP, the region still maintains nationally leading acreage and yield of *Malus domestica* and *Pyrus pyrifolia*, offering a representative setting for examining the distribution patterns and driving factors of fruit-tree richness in North China.

### 2.2 Data

#### 2.2.1 Distribution data of major fruit trees

When using the MaxEnt model for species distribution simulation, the number and representativeness of sample points have an important impact on the model results. The MaxEnt model can consider the interactions and nonlinear relationships among variables. If the distribution points are too few, these complex relationships may not be fully captured, and model overfitting may occur (Wisz et al., 2008). The sampling points selected in this study are mainly distributed in fruit tree planting areas or orchards of certain scale, so as to better represent the actual distribution pattern of fruit trees in the region; the number of collected species distribution points meets the minimum requirement of the MaxEnt model (at least 5), and the number of distribution points for a single fruit tree species reaches up to 416, ensuring that the samples are both moderate in number and representative (Phillips et al., 2006). Fruit tree distribution data mainly come from public databases and literature sources. Among them, *Punica granatum* (43 sample points), *Ficus carica* (25 sample points), crab*Malus domestica* (49 sample points), *Crataegus pinnatifida* (166 sample points), *Prunus persica* (65 sample points), and *Castanea mollissima* (115 sample points) come from the Plant Science Data Center (https://www.plantplus.cn/cn); *Prunus avium* distribution data come from the *China Fruit Resource Map*; *Ziziphus jujuba* (791 sample points), *Malus domestica* (416 sample points), *Pyrus pyrifolia* (326 sample points), *Juglans regia* (135 sample points), *Diospyros kaki* (42 sample points), and *Corylus heterophylla* (321 sample points) come from the *Vegetation Map of China* (1:1,000,000) (https://www.plantplus.cn/dsite/zhibei/b12.html) (**Table 1**). By searching the Latin name or Chinese name of the tree species, specific distribution points were obtained, and their latitude and longitude information was extracted to generate species distribution data.

**Table 1:** Fourteen major fruit tree species in North China.

Species name	Family	Genus	Number of sample points
*Castanea mollissima*	Fagaceae	*Castanea*	115
*Corylus heterophylla*	Betulaceae	*Corylus*	321
*Crataegus pinnatifida*	Rosaceae	*Crataegus*	166
*Diospyros kaki*	Ebenaceae	*Diospyros*	42
*Ficus carica*	Moraceae	*Ficus*	25
*Juglans regia*	Juglandaceae	*Juglans*	135
*Malus domestica*	Rosaceae	*Malus*	416
*Malus spectabilis*	Rosaceae	*Malus*	49
*Prunus armeniaca*	Rosaceae	*Prunus*	36
*Prunus avium*	Rosaceae	*Prunus*	36
*Prunus persica*	Rosaceae	*Prunus*	65
*Punica granatum*	Lythraceae	*Punica*	43
*Pyrus pyrifolia*	Rosaceae	*Pyrus*	326
*Ziziphus jujuba*	Rhamnaceae	*Ziziphus*	791

#### 2.2.2 Driving factors data

When simulating the richness of major fruit trees using the MaxEnt model, the climate data used in this study were obtained from the WorldClim database (http://worldclim.org) (Fick and Hijmans, 2017), selecting current climate data (with a resolution of 2.5′, approximately corresponding to a spatial accuracy of 3.5–4.6 km). Terrain data were obtained from the SRTM DEM 90 m (van Zyl, 2001), and resampled to ensure consistency with the factor grids. Soil data were obtained from the 2023 global soil dataset HWSD2.0 (Harmonized World Soil Database 2.0), used to analyze the influence of soil factors on fruit tree richness. Socioeconomic variables included Gross Domestic Product (GDP), population density (POP), night-time light intensity (NTL), and road density (RD). GDP and NTL data were obtained from the Resource and Environment Science and Data Center of the Chinese Academy of Sciences, based on the *China GDP Spatial Distribution Kilometer Grid*.

*Dataset* and the *China Night-Time Light Annual Dataset*, respectively. Population density data were sourced from the WorldPop database. Road density data were derived from the *2019 China 1 km Grid Road Density Dataset* provided by the Science Data Bank (**Table 2**).

**Table 2:** Driving factors used for studying the distribution patterns of major fruit trees in North China.

Driving factors		Description	Unit
Climate Variables	AMT	Annual Mean Temperature	°C
	MDR	Mean Diurnal Range (Mean of monthly (max temp - min temp)	°C
	ISOT	Isothermality (BIO2/BIO7) (×100)	%
	TS	Temperature Seasonality (standard deviation ×100)	°C
	MTWM	Max Temperature of Warmest Month	°C
	MTMC	Min Temperature of Coldest Month	°C
	TAR	Temperature Annual Range (BIO5-BIO6)	°C
	MTWQ	Mean Temperature of Wettest Quarter	°C
	MTDQ	Mean Temperature of Driest Quarter	°C
	MTWAQ	Mean Temperature of Warmest Quarter	°C
	MTCQ	Mean Temperature of Coldest Quarter	°C
	AP	Annual Precipitation	mm
	PWM	Precipitation of Wettest Month	mm
	PDM	Precipitation of Driest Month	mm
	PSE	Precipitation Seasonality (Coefficient of Variation)	%
	PWQ	Precipitation of Wettest Quarter	mm
	PDQ	Precipitation of Driest Quarter	mm
	WQP	Warmest Quarter Precipitation	mm
	PCQ	Precipitation of Coldest Quarter	mm
Soil Variables	TBS	Topsoil Base Saturation	%
	TCC	Topsoil Calcium Carbonate	%
	TOC	Topsoil Organic Carbon	g kg^-1^
	TP	Topsoil PH(H_2_O)	
Terrain Variables	ASP	Aspect	°
	ELEV	Elevation	m
	SLP	Slope	°
Anthropogenic Variables	GDP	Gross Domestic Product	10^4^ CNY / pixel
	NTL	Night-Time Light (intensity)	digital number (DN)
	POP	Population Density	capita per grid cell
	RD	Road Density	km·km^−^²

### 2.3 Methods

#### 2.3.1 Preprocessing of driving factor data

This study incorporated 19 bioclimatic variables from WorldClim, 3 terrain variables derived from the SRTM DEM, 4 soil variables from the 2023 HWSD2.0 dataset, and 4 socioeconomic variables representing human activities to model and analyze fruit tree richness, with all variables resampled to a unified spatial resolution.

To ensure compatibility of data in different formats and smooth import into niche modeling software, the Catalog function in ArcGIS 10.2 was used, and the Raster to ASCII operation in the Basic Tools of the SDMToolbox v2.5 toolbox was employed to convert the downloaded raster-format climate data into ASC format, ensuring compatibility and processability of the data in subsequent analyses.

**Figure 2:**
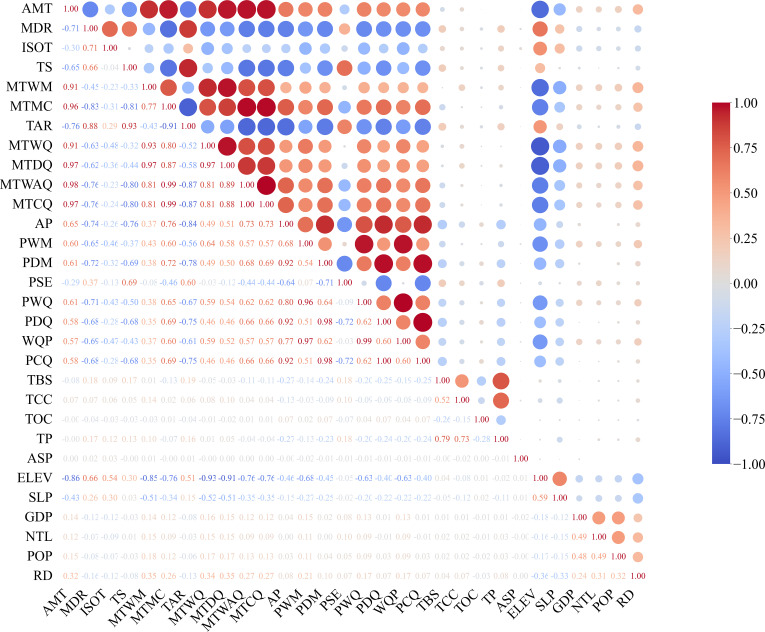
Correlation heatmap of factors.

To facilitate the import of fruit tree distribution point data into the MaxEnt model for analysis, the distribution point data were converted to CSV format using Excel. After importing all variables and the distribution point data of 14 fruit tree species into the MaxEnt model, preliminary simulations were conducted to calculate the contribution of each environmental variable to each species, and variables with cumulative contributions exceeding 95% were selected. For each species, variables were first ranked according to their contribution, and those cumulatively accounting for the top 95% of total contribution were retained. Subsequently, the occurrence frequency of each variable was calculated across all species, defined as the number of species in which the variable was included in the top 95% cumulative contribution set. Variables with zero occurrence frequency (i.e., not selected in the top 95% cumulative contribution for any species) were excluded from further analysis. For the remaining 26 environmental variables, correlation analysis was performed. If the absolute value of the correlation coefficient between two variables was greater than 0.8, only the variable with a higher occurrence frequency in the initial simulation was retained, in order to avoid the influence of multicollinearity on the model results (**Figure 2**). The final retained factors are shown in **Table 3**.

**Table 3:** Final driving factors selected in this study.

Driving factors		Description	Unit
Climate Variables	AMT	Annual Mean Temperature	°C
	MDR	Mean Diurnal Range (Mean of monthly (max temp - min temp)	°C
	ISOT	Isothermality (BIO2/BIO7) (×100)	%
	TS	Temperature Seasonality (standard deviation ×100)	°C
	PWM	Precipitation of Wettest Month	mm
	PSE	Precipitation Seasonality (Coefficient of Variation)	%
Soil Variables	TBS	Topsoil Base Saturation	%
	TCC	Topsoil Calcium Carbonate	%
	TOC	Topsoil Organic Carbon	g kg^-1^
	TP	Topsoil PH(H_2_O)	
Terrain Variables	ASP	Aspect	°
	ELEV	Elevation	m
	SLP	Slope	°
Anthropogenic Variables	GDP	Gross Domestic Product	10^4^ CNY / pixel
	NTL	Night-Time Light (intensity)	digital number (DN)
	POP	Population Density	capita per grid cell
	RD	Road Density	km·km^−^²

#### 2.3.2 Principle of the MaxEnt model

In ecology, the MaxEnt model is commonly used for species distribution prediction, analyzing the relationship between environmental factors and species distribution to help predict potential habitats of species. The MaxEnt model has significant advantages in dealing with uncertainty, especially under conditions of incomplete information. By maximizing entropy, the model can generate a probability distribution that best fits the existing information under known constraints, and can avoid making excessive assumptions when data are insufficient by minimizing assumptions, thereby reducing model bias.

Information entropy is an indicator that measures the uncertainty of a random variable, defined as:

(1) $$H(\xi )=-\sum_{i-1}^{n}P_{i}logP_{i}\;$$

In Equation 1, ${\text{P}}_{\text{i}}$is the probability that the random variable takes the *i*-th value. The larger the entropy, the higher the uncertainty of the system; the smaller the entropy, the more certain the system. The core idea of the maximum entropy principle is to select the probability distribution with the largest entropy under known constraints. Suppose that some constraints are known in this study, such as the expected value of the random variable. The study needs to maximize entropy under these constraints. Suppose there are n possible states, and the probability of each state is P_i_. The study needs to maximize the sum of probabilities equal to 1 under the following constraints: $\sum_{\text{i}=1}^{\text{n}}{\text{P}}_{\text{i}}\;=1$, and satisfy the known expectation constraints $\;\sum_{\text{i}=1}^{\text{n}}{\text{P}}_{\text{i}}{\text{x}}_{\text{ij}}\;={\text{M}}_{\text{j}}$,where $x_{ij}$ is the j**-**th feature value of the i-th state. Equation 2 shows the objective function (Sun et al., 2021) becomes:

(2) $$L=-\sum_{i=1}^{n}P_{i}\log P_{i}+\lambda_{0}\;(\sum_{i=1}^{n}P_{i}-1)+\sum_{j=1}^{m}\lambda_{j}\;(\sum_{i=1}^{n}P_{i}x_{ij}-M_{j}\;)$$

By taking the derivative of ${\text{P}}_{\text{i}}$ and setting the derivative equal to 0: $\frac{\partial L}{\partial f(x)}=-lnf(x)-1+\lambda_{0}+1+\sum_{i=1}^{m}\lambda_{i}x_{i}\;=0$, Solving yields $\;f(x)=\text{exp}(\lambda_{0}+\sum_{i=1}^{m}\lambda_{i}x_{i})$. Since the sum of probabilities is 1, the formula can be further simplified to Equation 3, where $Z=\sum_{k=1}^{n}exp(\sum_{j=1}^{m}\lambda_{j}x_{kj})\;$ is the normalization constant (Levine, 2025).

(3) $$f(x)=\frac{\exp(\sum_{i=1}^{m}\lambda_{i}x_{i})}{Z}$$

The MaxEnt maximum entropy model belongs to a type of ecological niche model. It can predict suitable habitats of species under limited species distribution data while maintaining high accuracy. Phillips et al., based on the maximum entropy principle, implemented the MaxEnt model in JAVA and analyzed the dominant environmental factors affecting plant distribution using linear regression (i.e., the Jackknife method). This allows prediction map generation, ROC curve accuracy analysis, and dominant environmental factor analysis to be automatically completed by the MaxEnt model, greatly improving the model’s application efficiency. The MaxEnt 3.4.4 software used in this study was downloaded from https://BIOdiversityinformatics.amnh.org/open_source/maxent/.

#### 2.3.3 Model accuracy evaluation method

The simulation accuracy of the MaxEnt model is usually evaluated by the area under the receiver operating characteristic (ROC) curve (AUC value), whose magnitude reflects the model’s ability to distinguish between areas of species presence and absence (Hanley and McNeil, 1982). The AUC value ranges from 0 to 1, where 0.5 indicates that the model’s discriminative ability is equivalent to random guessing, and 1 indicates perfect discrimination. An AUC value between 0.5 and 0.7 indicates low discriminative ability, which may not provide very reliable estimates of suitable habitats; between 0.7 and 0.9 indicates moderate discriminative ability; and above 0.9 indicates high accuracy and credibility, providing a reliable basis for species distribution research and management.

The standard deviation can be used to measure the stability of the model simulation results. The smaller the value, the more consistent the model outputs. As shown in the table, most species have small standard deviations, indicating that the model’s simulation results for the current suitable habitats of fruit trees are relatively stable.

#### 2.3.4 Definition of richness

To binarize the habitat suitability probabilities predicted by the MaxEnt model, we applied the natural breaks classification method, which minimizes within-class variance and maximizes between-class variance, thus identifying intrinsic patterns in the data rather than imposing arbitrary intervals. This approach is particularly suitable for environmental modeling outputs, where suitability scores are often skewed or clustered (Ghanbari et al., 2025). The Jenks Natural Breaks method has been used in species distribution modeling (Tian et al., 2025).For each fruit tree’s suitable probability TIFF file, the natural breaks classification method was first used to determine key breakpoints (Stockwell and Peterson, 2002), and binarization was performed accordingly:

(4) $$B_{i}(x)=\{\begin{array}{cc} 1, & P_{i}(x)>T_{i}\\ 0, & P_{i}(x)\leq T_{i} \end{array}$$

In Equation 4, $P_{i}(x)$ represents the suitability probability of fruit tree i at pixel  x, $T_{i}$ is its natural breakpoint threshold, $\;B_{i}(x)=1$ indicates that pixel x belongs to the suitable growth area of fruit tree  i, and $B_{i}(x)=0$ indicates an unsuitable area (Jiang, 2013; Wang et al., 2024). Subsequently, the binarized rasters of the 14 fruit tree species were overlaid to obtain the richness distribution:

(5) $$R(x)=\sum_{i=1}^{n}B_{i}(x)$$

In Equation 5  ,  R(x) represents the number of fruit tree species suitable for growth at pixel  x, i.e., fruit tree richness;*n =*14.

#### 2.3.5 Geodetector-based Interaction analysis

To further investigate how different categories of environmental and anthropogenic factors jointly influence the spatial distribution of fruit tree richness, this study introduced the interaction module of Geodetector to compare the explanatory power of individual driving factors and their combinations on fruit tree richness (Wang et al., 2010). Interaction detection was first performed between pairs of individual factors to quantify their two-factor interaction effects.

Specifically, driving factors were reclassified into four groups: climate (AMT, MDR, ISOT, TS, PWM, PSE), soil (TBS, TCC, TOC, TP), terrain (SLP, ASP), and anthropogenic (GDP, NTL, POP, RD). Within each group, multiple factors were jointly combined to represent group-level effects, and interaction strengths were further calculated both within and across the four factor groups.

By integrating interaction detection at the individual-factor level and the factor-group level, this approach distinguishes the detailed interactions among specific variables from the integrated synergistic effects among major factor categories, thereby identifying the dominant driving factor groups and their combined effects on fruit tree distribution patterns, and providing a basis for understanding the multi-factor driving mechanisms.

#### 2.3.6 Structural equation model construction

To reveal the mechanisms by which environmental and anthropogenic factors affect fruit tree richness, this study introduced SEM for analysis. SEM integrates the concepts of factor analysis and path analysis, allowing simultaneous handling of relationships between latent variables and observed variables, and characterizing both direct and indirect effects among latent variables. Its general form includes two parts: the measurement model (Equation 6) and the structural model (Equation 7):

(6) $$X=\Lambda_{x}\xi +\delta ,Y=\Lambda_{y}\eta +\epsilon$$

(7) η=Bη+Γξ+ζ

where ξ represents exogenous latent variables,  η represents endogenous latent variables, X and Y are the matrices of observed variables, $\;\Lambda_{x}\text{ and }\Lambda_{y}$ are factor loading matrices,  δ and ϵ are measurement errors, and  ζ is the structural error.

In this study, terrain (T), climate (C), soil (S) and anthropogenic (A) were treated as latent variables, represented by their corresponding indicators, and fruit tree richness (R) was treated as the endogenous variable. In this framework, terrain and anthropogenic factors were specified as exogenous variables, influencing climate and soil but not being affected by them, as the model focuses on ecological timescales where feedback effects (e.g., climate-driven terrain change) are negligible. According to ecological mechanisms, the path relationships were set as follows:

(8) $$R=\beta_{1}T+\beta_{2}C+\beta_{3}S+\beta_{4}A+\epsilon$$

(9) $$C=\gamma_{1}A+\gamma_{2}T+\zeta_{1}$$

(10) $$S=\gamma_{3}A+\gamma_{4}T+\zeta_{2}$$

In Equations 8-10, $\;\beta_{1},\beta_{2},\;\beta_{3}\text{and}\;\beta_{4}\;$represent the path coefficients of terrain, climate, soil and human activity on fruit tree richness; $\;\gamma_{1},\gamma_{2},\;\gamma_{3}\;and\;\gamma_{4}$ represent the path coefficients of exogenous variables (terrain and anthropogenic factors) on soil and climate; $\;\epsilon ,\zeta_{1}\text{ and }\zeta_{2}$ are random error terms.

#### 2.3.7 Technical route

**Figure 3:**
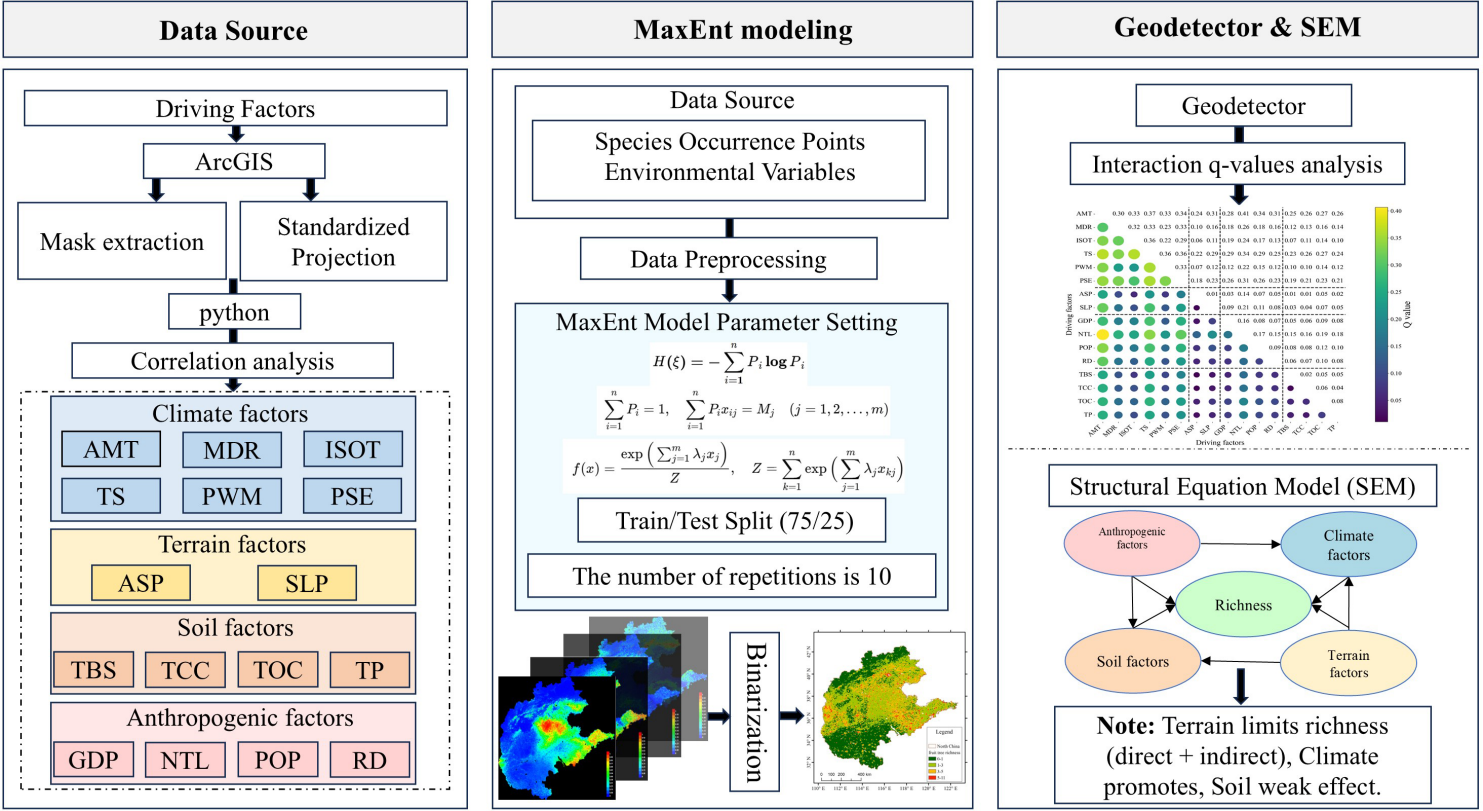
Schematic illustration of the proposed strategy for evaluating the distribution patterns and driving factors of fruit tree richness in North China.

The proposed strategy is visually represented in **Figure 3**. The research results will help to identify the key driving factors affecting fruit tree richness distribution, to understand the driving mechanisms of multiple factors on fruit tree richness, and to provide scientific basis for the optimization of fruit tree planting layout and regional management in North China.

## 3 Results analysis

### 3.1 Model accuracy evaluation

To simulate the potential suitable habitats of major fruit trees in Northern China, this study imported the collected fruit tree distribution data (in CSV format) and 16 factor datasets into MaxEnt 3.4.4 for modeling. During the computation, 75% of the data were used as the training dataset and 25% as the testing/validation dataset, with the number of training repetitions set to 10 (Zhang et al., 2021) to achieve the simulation of current suitable habitats.

**Figure 4:**
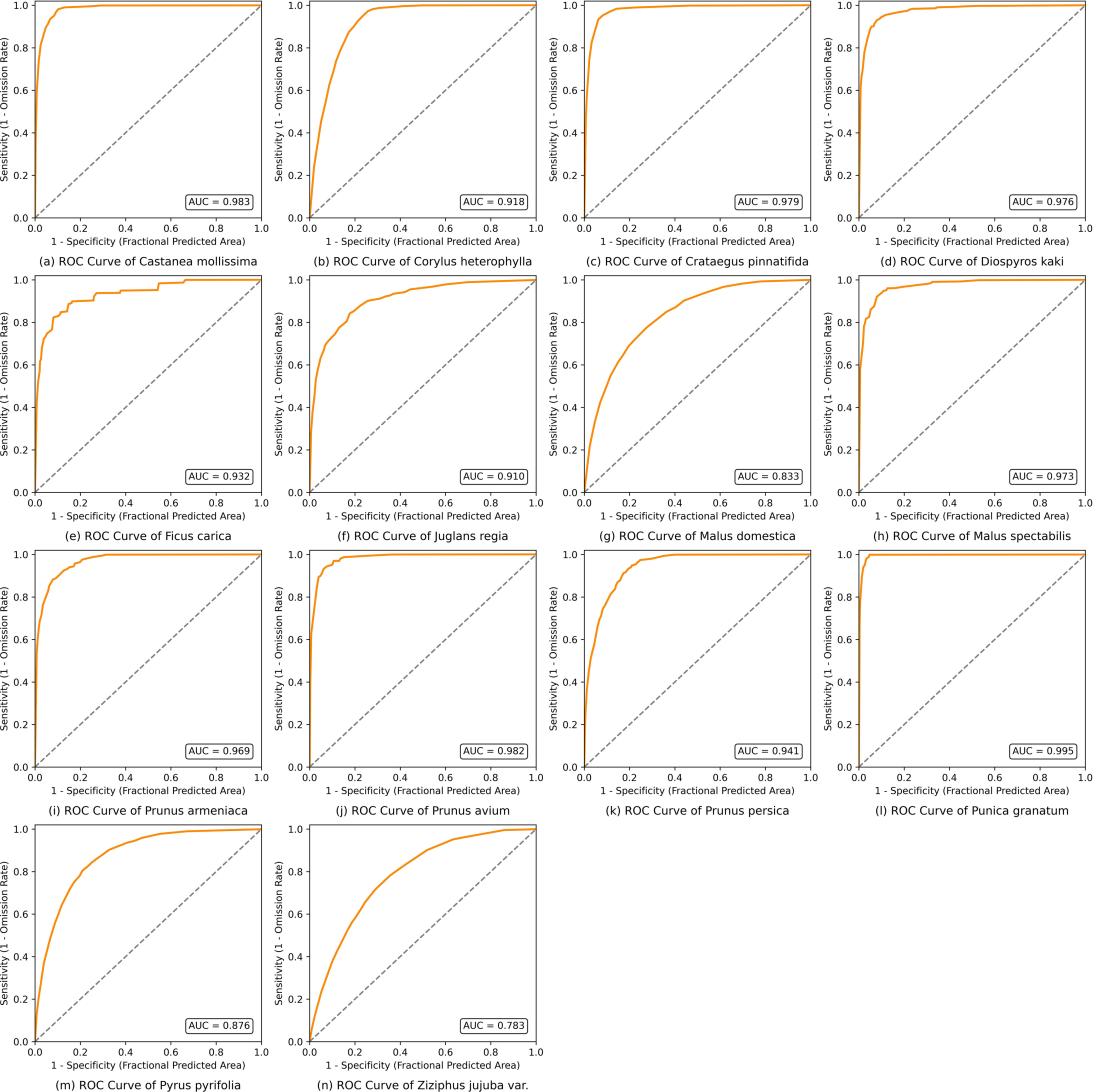
ROC curves of major fruit trees in North China.

The model accuracy in this study was evaluated using the ROC curve and AUC value. The AUC value reflects the model’s ability to distinguish areas of species presence from absence, ranging from 0 to 1, with higher values indicating higher simulation accuracy (Elith et al., 2010). As shown in **Table 4**, the AUC values of most fruit tree species exceeded 0.8, indicating high simulation accuracy and reliable results under current climate conditions. The three highest values were recorded for *Punica granatum* (0.995), *Castanea mollissima* (0.983), and *Prunus avium* (0.982), showing that the model achieved extremely high accuracy in simulating the suitable habitats of these species. Except for *Ziziphus jujuba* (0.783), whose AUC values were slightly below 0.8, all other species had AUC values above 0.8, meeting the modeling requirements (**Figure 4**). In summary, the AUC values of the MaxEnt model in this study are consistent with the simulation accuracy reported in previous studies (Grenié et al., 2020; Stas et al., 2020), where most literature reported AUC values above 0.8 (Xiao et al., 2024; Khwarahm, 2025).

**Table 4:** MaxEnt model simulation accuracy (AUC) of fruit trees in North China.

Species name	Mean training AUC	Standard deviation
*Castanea mollissima*	0.983	0.004
*Corylus heterophylla*	0.918	0.004
*Crataegus pinnatifida*	0.979	0.003
*Diospyros kaki*	0.976	0.013
*Ficus carica*	0.932	0.044
*Juglans regia*	0.910	0.014
*Malus domestica*	0.833	0.008
*Malus spectabilis*	0.973	0.008
*Prunus armeniaca*	0.969	0.008
*Prunus avium*	0.982	0.005
*Prunus persica*	0.941	0.010
*Punica granatum*	0.995	0.002
*Pyrus pyrifolia*	0.876	0.010
*Ziziphus jujuba*	0.783	0.010

### 3.2 Current fruit tree richness in North China

In order to explore the distribution of major fruit tree richness in North China, this study first constructed potential habitat distribution maps for each species using the MaxEnt model. Based on these results, the suitable habitat areas were reclassified and extracted. Subsequently, the habitat rasters of 14 fruit tree species were overlaid to generate a spatial distribution map representing fruit tree species richness.

**Figure 5:**
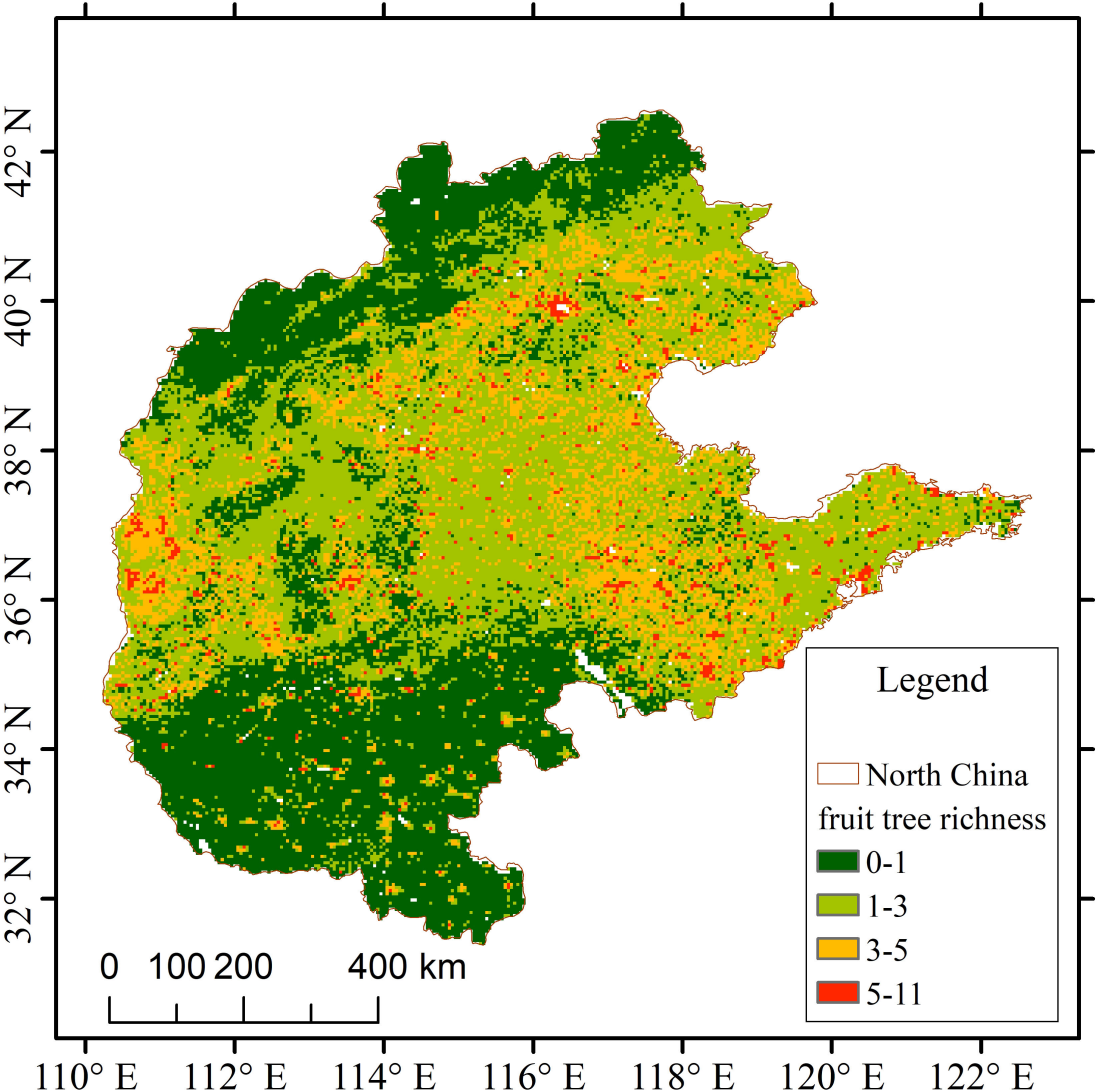
The current distribution of fruit tree richness for 14 species in North China.

The spatial distribution of fruit tree richness in the North China region exhibits clear spatial heterogeneity (**Figure 5**). High-richness areas (red) are mainly concentrated along the eastern Taihang Mountains, the Shandong Hills, and the Jiaodong Peninsula, forming relatively continuous clusters. Medium-richness areas (yellow) are distributed across eastern Shanxi, central Hebei, and western Shandong, while low-richness areas (yellow–green) dominate the extensive plains. Non-suitable zones (dark green) are mainly located Henan Province.

When combined with the geographical reference map (**Figure 1**), high-richness areas not only correspond to hilly and mountainous landscapes but also show clear spatial overlap with regions of intensive human activity, particularly in long-established agricultural and orchard-dominated zones. To further quantify this pattern, correlations between fruit tree richness and all environmental variables were calculated at the pixel level. The results show that anthropogenic variables are consistently positively correlated with richness, and overall rank among the higher positions compared to other factors, with night-time light (NTL, r = 0.28759), population density (POP, r = 0.21635), road density (RD, r = 0.18293), and GDP (r = 0.14855) showing relatively strong associations. In contrast, plains with extensive cultivation but relatively simplified land-use structures tend to exhibit lower richness. Overall, fruit tree richness displays a distinct spatial gradient shaped by topography and climate, while being strongly modulated by human land-use patterns.

### 3.3 Key driving factors influencing the distribution of major fruit tree diversity

This study collected distribution data for 14 fruit tree species and employed the MaxEnt model to simulate the potential suitable habitats of each species, while calculating the contribution rate of each ecological factor to the simulation results. For each species, MaxEnt ranks the ecological factors according to their contribution to habitat suitability modeling. Given the large number of fruit tree species involved, listing the contribution rankings of ecological factors for all 14 species is insufficient to comprehensively evaluate their overall influence. Therefore, a statistical approach was adopted to determine the relative importance of each ecological factor: the frequency with which an ecological factor ranked among the top three contributors (largest, second largest, or third largest) across all species was calculated and then ranked. This reflects the overall importance of ecological factors in modeling fruit tree habitat suitability (Lai et al., 2024).

**Figure 6:**
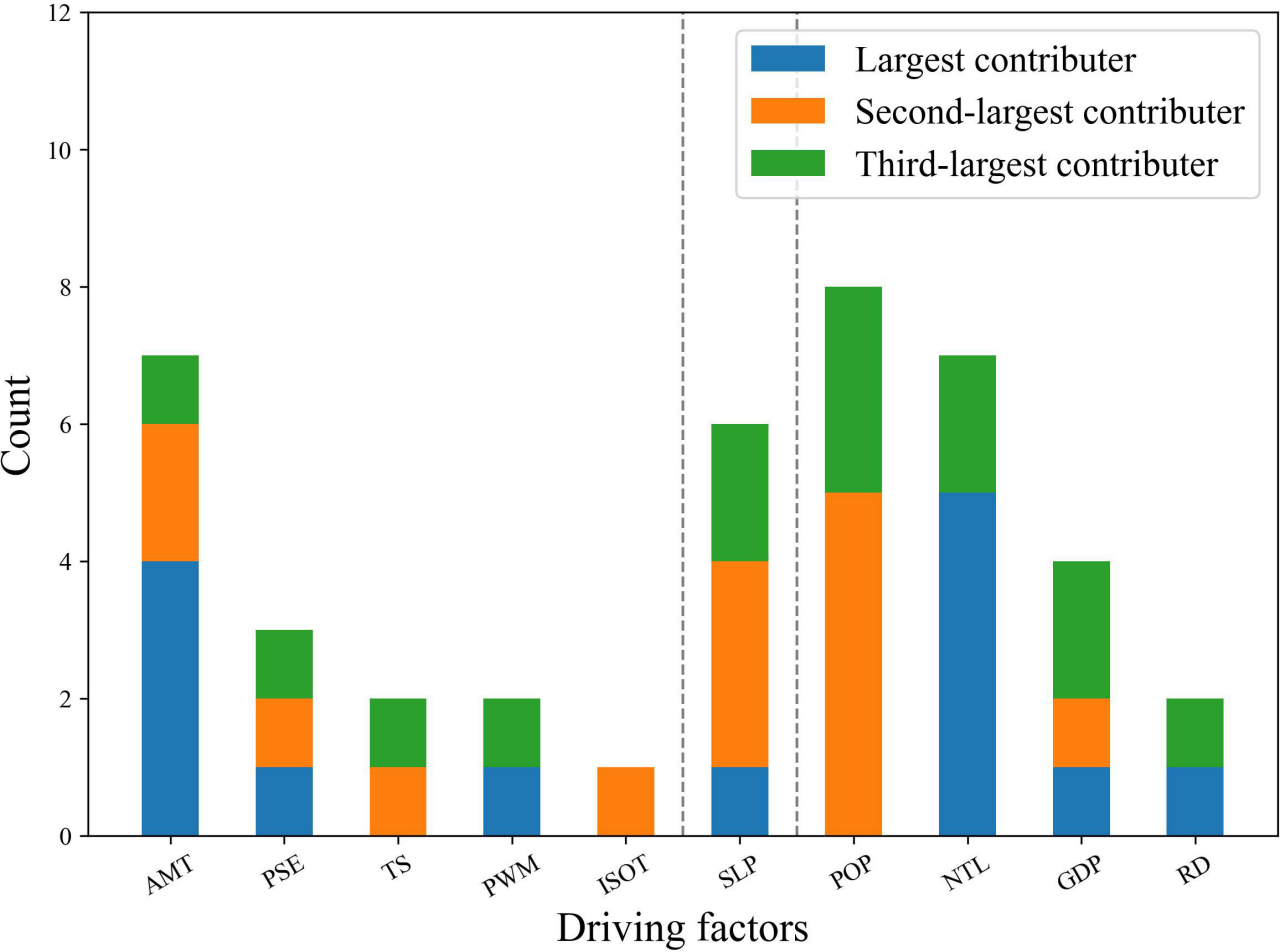
Statistical ranking of driving factor contributions in the MaxEnt model for major fruit trees in the North China region. The y-axis (“Count”) represents the number of fruit tree species (out of 14) for which a given ecological factor ranked among the top three contributors to MaxEnt habitat suitability modeling.

Under current climate conditions, the ensemble modelling of the fourteen fruit-tree species reveals a clear hierarchy among ecological variables. Population density most frequently appears among the top three contributors, followed closely by annual mean temperature and night-time light intensity; slope ranks next, while gross domestic product and precipitation seasonality occupy intermediate positions, and remaining factors seldom reach the upper ranks. Anthropogenic variables, especially population density and night time light intensity, consistently dominate the contribution structure, underscoring the strong influence of anthropogenic pressure on fruit tree richness (**Figure 6****).**

### 3.4 Analysis of the interaction characteristics of driving factors

**Figure 7:**
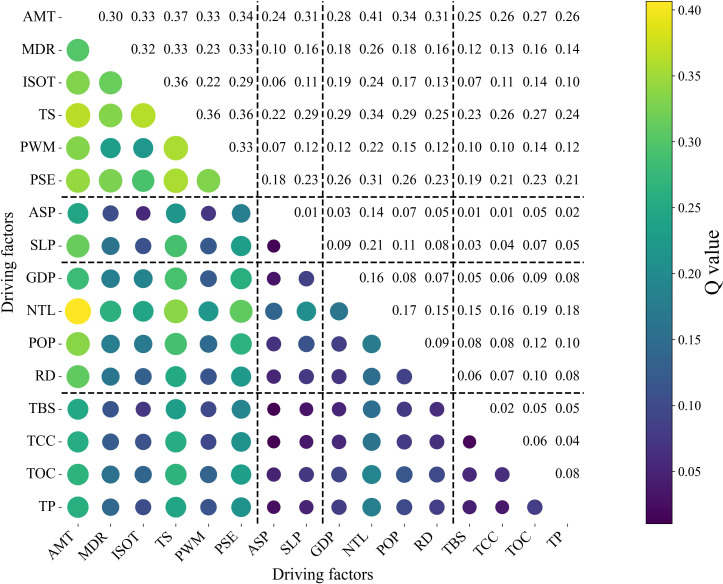
Interaction matrix of driving factors (Q values).

According to **Figure 7**, interaction Q values among different factors show clear contrasts. Interactions involving climate variables generally exhibit higher Q values, especially when coupled with anthropogenic factors. For example, the interaction between AMT and NTL reaches 0.4066, representing the highest interaction strength among all factor pairs. TS also shows consistently high interaction Q values with multiple factors, such as AMT (0.3656), ISOT (0.3627), and PWM (0.3570). In contrast, interactions within terrain factors are relatively weak; for instance, the interaction between ASP and SLP is only 0.0126.

As shown in **Table 5**, the average interaction Q values differ markedly among factor combinations. Climate–anthropogenic interactions are the strongest (0.9770), followed by climate–terrain (0.8600) and climate–soil (0.8003). Terrain–anthropogenic (0.7757) and anthropogenic–soil interactions (0.7421) show moderate values. In comparison, interactions within the same factor groups are much lower, particularly within-terrain (0.0126) and within-soil factors (0.0913).

**Table 5:** Average interaction Q-values of different factor combinations.

Factor combination	Average interaction Q-value
Climate + Anthro	0.9770
Climate + Terrain	0.8600
Climate + Soil	0.8002
Terrain + Anthro	0.7756
Anthro + Soil	0.7420
Within-climate factors	0.6140
Within-anthro factors	0.3331
Within-soil factors	0.0912
Soil + Terrain	0.1901
Within-terrain factors	0.0126

Overall, interaction effects vary substantially across factor combinations, with higher Q values mainly associated with interactions involving climate and anthropogenic factors, while intra-terrain and intra-soil interactions remain weak.

### 3.5 Multi-factor pathway mechanisms driving fruit tree richness

To further disentangle how natural and anthropogenic forces jointly shape plant richness, the 16 original variables were synthesized into four latent blocks—terrain, climate, soil, and human activity—and a structural equation model was fitted. Considering the very large sample size (n = 39,453), the overall fit indices are slightly below the conventional thresholds but still reasonable: CFI = 0.8892, GFI = 0.8889, NFI = 0.8889. These values, being close to 0.9, indicate that the model provides an acceptable approximation of the observed data. Importantly, all hypothesized paths show strong effects, with every path was significant at p < 0.001.

**Figure 8:**
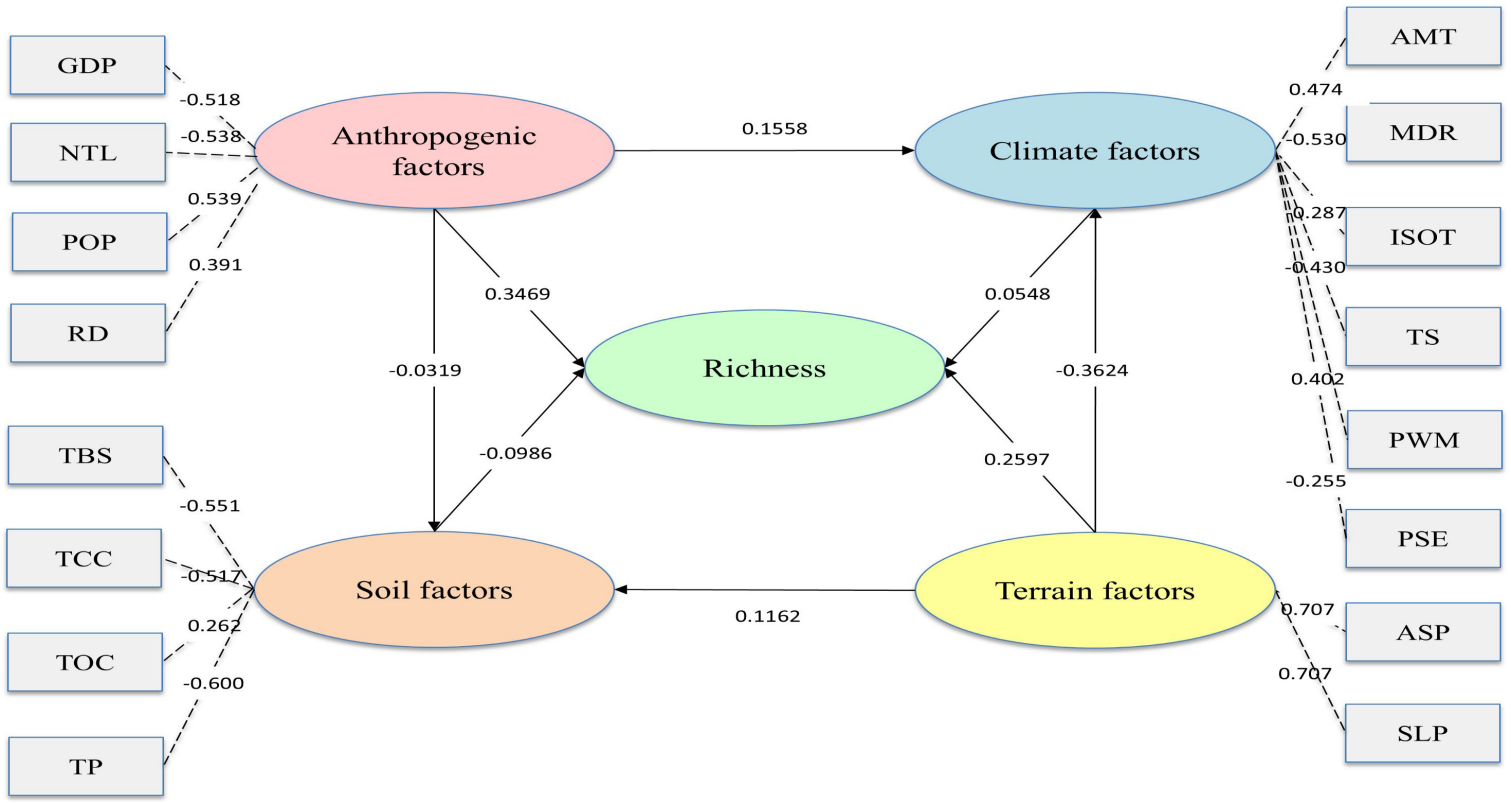
Effect of terrain, climate, soil and anthropogenic factors on fruit tree richness revealed by structural equation modeling. Standardized path coefficients are displayed along the arrows. All path coefficients are highly significant at p < 0.01. Standard errors and 95% confidence intervals are reported in **Table 6**. .

The standardized total effects on species richness mirror the sign and rank of the model estimates. According to **Figure 8**, anthropogenic factors show the strongest association with plant richness (0.3469), exceeding all natural factors, with weak positive indirect effects via climate (0.1558) and soil (−0.0319). Human activities are weakly associated with variations in climatic factors such as temperature and precipitation, though this effect remains marginal. Terrain follows (0.2597), but its negative association with climate (−0.3624) and positive association with soil (0.1162)—which itself is negatively associated with richness (−0.0986)—attenuate its net contribution. Climate shows a modest direct effect (0.0548), while soil is negatively associated with richness. Indirect pathways remain marginal and are dwarfed by direct effects. Overall, human activity is identified as the most strongly associated factor, followed by terrain, with climate and soil playing minor or suppressive roles.

**Table 6:** Standardized path coefficients and 95% confidence intervals in the structural equation model.

Path	Standardized coefficient (β)	95% CI
Climate index ← Terrain index	-0.3624	[-0.3798, -0.3450]
Climate index ← Anthro index	0.1558	[0.1440, 0.1675]
Soil index ← Terrain index	0.1162	[0.1006, 0.1319]
Soil index ← Anthro index	-0.0319	[-0.0425, -0.0213]
Richness ← Terrain index	0.2597	[0.2451, 0.2742]
Richness ← Soil index	-0.0986	[-0.1075, -0.0896]
Richness ← Anthro index	0.3469	[0.3371, 0.3566]
Richness ← Climate index	0.0548	[0.0467, 0.0628]

## 4 Discussion

### 4.1 Model performance and predictive reliability

The MaxEnt model applied in this study is based on the principle of maximum entropy, which can handle both continuous and categorical variables under small sample conditions and account for nonlinear relationships among factors. It has been widely used in predicting the potential suitable habitats of multiple species (Li et al., 2020, 2022). We developed simulation models for the potential suitable areas of 14 fruit tree species, and the AUC values indicate that the predictive accuracy for most species exceeded 0.8, with only *Crataegus pinnatifida* and *Ziziphus jujuba* showing slightly lower AUC values.

The relatively low AUC values for *Crataegus pinnatifida* and *Ziziphus jujuba* occupy diverse habitats, including farmland margins, hillside shrublands, and secondary forests. Niche breadth negatively affects SDM performance. Species with narrower niches and smaller occurrence areas tend to yield higher predictive accuracy. The weak environmental constraints and diffuse distribution boundaries of generalist species reduce the discriminative power of models, resulting in less precise predictions of suitable habitats (Tessarolo et al., 2021). The consistent ecological characteristics of these two species support this interpretation.

### 4.2 Analysis of the driving factors of fruit tree richness distribution

Based on the results presented above, in the North China region, the spatial pattern of fruit tree richness is associated with multiple factors, including climate, terrain, soil and human activity. High-richness areas are mainly concentrated in the eastern Taihang Mountains, the southern part of Shanxi Province, the Shandong Hills, and Jiaodong Peninsula, where suitable temperature and moisture conditions coincide with hilly land conducive to orchard establishment (Peng et al., 2020). In the typical terrain of the eastern and western flanks of the Taihang Mountains, windward slopes (north/northeast-facing) are affected by proximity to the Bohai Bay and maritime climate, resulting in abundant summer moisture, moderate solar radiation, and sufficient water supply, which support higher plant richness. In contrast, leeward slopes (west-facing) lie in the rain shadow of the summer monsoon, where precipitation is significantly reduced and water limitation is intensified, leading to comparatively lower fruit tree richness (Zeng et al., 2014).

The strongest interaction detected between climate and anthropogenic factors (Q = 0.9770) indicates that neither driver operates independently in shaping fruit tree richness. This suggests that terrain-mediated climatic heterogeneity plays a critical role in shaping fruit tree distribution, as topographic factors regulate local temperature, moisture availability, and radiation conditions, thereby influencing habitat suitability. The SEM results further provide insight into the pathways underlying this interaction. As shown in **Table 5**, this interaction is considered comparatively strong due to its high Q value, which reflects substantial explanatory power relative to other factor combinations. While climate exerts a statistically significant direct effect on fruit tree richness (0.0548), its explanatory strength is substantially weaker than that of anthropogenic factors, which show the largest direct positive effect (0.3469). More importantly, anthropogenic factors also are linked to fruit tree richness through indirect pathways involving climatic variables, indicating a coupled human–climate control rather than parallel effects (Rosenzweig et al., 2008). Variables such as GDP, night-time light intensity, population density, and road density collectively represent the intensity of human activities and economic development, which may be associated with variations in local thermal and moisture regimes through land-use change, urban expansion, and surface energy balance alterations (Rayeen et al., 2026). In North China, where highly urbanized metropolitan areas (e.g., Beijing and Tianjin) coexist with mountainous regions and rural counties, such contrasts are likely to strengthen the observed statistical pathways linking human activity and climatic conditions relevant to fruit tree growth.

The interaction between climate and terrain follows closely, with a Q value of 0.8600, highlighting their combined influence on fruit tree richness. Terrain shows a significant negative association with climate (-0.3624), whereas climate shows a positive direct effect on fruit-tree richness (0.0548). The indirect pathway from terrain through climate to richness therefore yields a small negative impact, indicating that terrain is associated with lower richness primarily through its relationship with primarily by diminishing local climatic favourability rather than enhancing it. As elevation increases, temperature decreases and precipitation decreases, corresponding to a reduced positive association between climate and on fruit tree distribution (Sang and Edward, 2001).

Overall, the results indicate that fruit tree richness in the North China region is shaped by interactions between climate and anthropogenic factors, as well as between climate and terrain. However, when these interactions are further disentangled, a clearer hierarchical structure emerges. Single-factor analysis indicates that anthropogenic factors are most strongly associated with fruit tree richness, followed by climate, with terrain playing a comparatively weaker role. In contrast, SEM highlights a pathway-based hierarchy in which anthropogenic factors show the strongest direct association with richness, while terrain ranks second by constraining climatic suitability, and climate acts primarily as an intermediate driver. Taken together, these results consistently identify anthropogenic factors as the dominant factor associated with fruit tree richness in the North China region. It should be noted that, as the study focuses on cultivated fruit tree species, the observed spatial patterns likely reflect a combination of ecological suitability and human management practices, rather than purely natural processes.

Different analytical approaches provide complementary perspectives on the drivers of fruit tree richness. The MaxEnt model indicates that population density is an important factor influencing fruit tree distribution, while the Geodetector highlights the role of interactions among factors. The structural equation model further shows that anthropogenic factors have the strongest direct effect on richness. These results suggest that no single method can fully capture the complexity of distribution patterns, and combining multiple approaches allows a clearer understanding of how terrain, climate, soil, and human activities jointly shape fruit tree richness.

### 4.3 Study limitations

First, the study mainly relied on occurrence data for large-scale cultivated fruit trees; for species with relatively few samples, such as *Ficus carica*, *Prunus armeniaca*, and *Prunus avium*, the reliability of the simulation results may be reduced. Particularly, differences in sample size and potential spatial sampling bias may affect the estimation of species’ environmental niches in the MaxEnt model, leading to over- or under-prediction of suitable habitats. When stacking individual species distributions to derive fruit tree richness, these biases may further propagate and introduce uncertainty into the richness estimation. In addition, the model validation was based on random data partitioning, which does not explicitly account for spatial autocorrelation in species occurrence data. This may lead to an overestimation of model performance, as nearby occurrence points are not fully independent. Furthermore, as aspect is typically discretized into several directional classes, this process may reduce the variability captured by the variable and weaken its explanatory power in interaction detection. Besides, we note that coupling MaxEnt with additional analytical frameworks, though appealing for dissecting complex causal pathways, carries the risk of artificially inflated explanatory power in theory.

Second, although the study covered the main fruit trees in North China, it did not include all species, which may affect the accuracy of local richness estimates. Moreover, MaxEnt predictions of suitable habitats often exceed the actual distribution ranges of species (Wan et al., 2019). Although this study explicitly incorporated anthropogenic factors, the representation of human activities was still simplified and may not fully capture fine-scale management practices and policy constraints. In reality, strict farmland protection policies in North China (e.g., permanent basic farmland regulations and land-use controls), land-use practices (Tolessa et al., 2017), irrigation conditions (Jafari et al., 2020), pest management, and species interactions (Levine et al., 2017) may all contribute to discrepancies between predicted suitability and actual fruit tree distributions.

Finally, this study assumed a temporally stable relationship between driving factors and species distributions, without fully accounting for potential shifts in fruit tree ecological niches under future climate change.

## 5 Conclusions

This study integrated the MaxEnt model with Geodetector and structural equation modeling (SEM) to investigate the spatial distribution patterns and driving mechanisms of fruit tree richness in the North China region, based on occurrence data of 14 major fruit tree species and a comprehensive set of climatic, terrain, soil, and anthropogenic variables. The main conclusions are as follows:

1. The MaxEnt model effectively simulated the potential suitable habitats of the 14 fruit tree species, with most species achieving AUC values above 0.8, indicating good predictive performance under current climatic conditions. Only *Ziziphus jujuba* showed slightly lower AUC values, likely due to its broad ecological niches and diffuse distribution boundaries.
2. High fruit tree richness in the North China region is mainly concentrated in hilly and mountainous areas such as the eastern Taihang Mountains, the Shandong Hills, and the Jiaodong Peninsula, while plains generally exhibit lower richness. This spatial pattern reflects the combined influence of terrain–climate gradients and intensive human land-use and orchard management.
3. Based on the MaxEnt ensemble results of 14 fruit tree species, population density emerges as the most important contributor to habitat suitability, followed by annual mean temperature and night-time light intensity, with slope also playing a notable role. Overall, human-related variables together with topographic factors dominate the spatial templates of fruit tree habitats in the North China region.
4. Geodetector results reveal strong interaction effects among driving factor combinations. Notably, climate–anthropogenic interactions show the highest explanatory power (Q = 0.9770) among all groups (**Table 5**), far exceeding interactions within terrain or soil factors. Structural equation modeling further confirms that anthropogenic factors exert the strongest direct positive effect on fruit tree richness (0.3469), followed by terrain, while climate plays a weaker positive role and soil mainly acts as a constraint. Collectively, the results suggest that fruit tree richness in the North China region is more strongly regulated by anthropogenic influences.

## Data Availability

The original contributions presented in the study are included in the article/Supplementary Material. Further inquiries can be directed to the corresponding author.
